# Science That Looks Like Me, That Looks Like Us

**DOI:** 10.1021/acscentsci.2c01340

**Published:** 2022-12-28

**Authors:** Miranda Currás Alcalá

The artwork featured on the cover of this issue of *ACS Central Science* was made to highlight the importance of diversity and representation in science. As a minority student myself, I was motivated to create a piece that made the observers say, “oh, that character looks just like me.” My goal was to develop an eye-catching design with a lot of vibrant colors and recognizable symbols from the represented groups, and from chemistry. I illustrated many organic forms and pursued a theme of nature and movement, capturing the beautiful diversity of humankind.

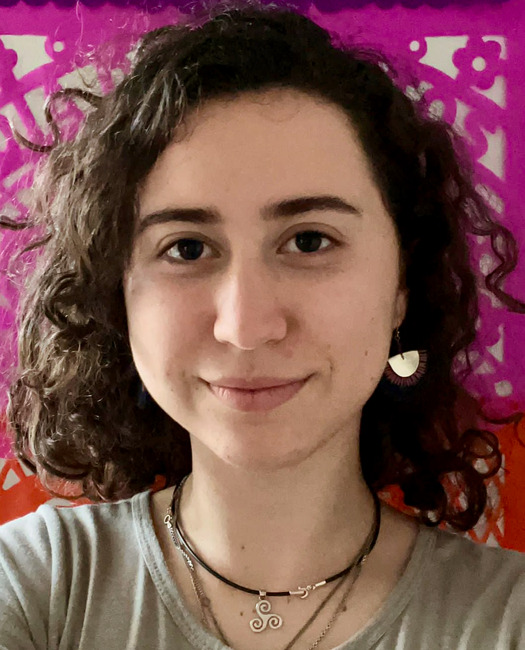



I grew up around a lot of scientists: my dad is a mathematician, my mother is a chemist, and my sister is a biotech engineer. Many of my direct relatives are also chemists and engineers. However, when growing up, whenever I read scientific articles or browsed those famous *Science* magazine covers, I never saw people like them, like us. With this cover piece, I got a chance to transform that experience by portraying a representation of all of us. I got to highlight how important diversity is to scientific and technological progress and how important we are, regardless of what we look like or where we come from.

When I read the call for invited cover submissions, the first idea that came to mind was to draw a table with a group of people from different backgrounds, coexisting together in the same space. I wanted to illustrate what a 21st century laboratory should look like, with a diverse group of people focused on the science, advancing knowledge through their unique perspectives, without their identity being put into question or into the spotlight. Each character in my cover has a relationship with the space where they appear and with the colleagues surrounding them.

Creating this cover has helped me a lot as an artist to experience a new way of thinking. I currently live as an immigrant in the US, in a city that is full of diversity and contrasts. My new home has taught me a new way of appreciating differences, of valuing artistic expression in any form, of identifying myself, and of interacting with others. Connecting with people through art can be difficult because one must be honest and respectful of the values and characteristics of those who are represented in the art piece. I hope that my art achieved that, and that every observer who identifies with this cover feels represented, empowered, and celebrated.

